# Cost-effectiveness analysis of overground robotic training versus conventional locomotor training in people with spinal cord injury

**DOI:** 10.1186/s12984-023-01134-7

**Published:** 2023-01-21

**Authors:** Daniel Pinto, Allen W. Heinemann, Shuo-Hsiu Chang, Susan Charlifue, Edelle C. Field-Fote, Catherine L. Furbish, Arun Jayaraman, Candace Tefertiller, Heather B. Taylor, Dustin D. French

**Affiliations:** 1 Department of Physical Therapy, College of Health Sciences, Marquette University, Milwaukee, USA; 2 Center for Rehabilitation Outcomes Research, Shirley Ryan AbilityLab, Chicago, USA; 3 Neurorecovery Research Center, TIRR Memorial Hermann, Houston, USA; 4 Craig Hospital, Englewood, USA; 5 Spinal Cord Injury, Shepherd Center, Atlanta, Georgia; 6 Shepherd Center, Atlanta, Georgia; 7 Max Näder Center for Rehabilitation Technologies and Outcomes Research and Outcomes Research, Shirley Ryan AbilityLab, Chicago, USA; 8 Research and Evaluation, Craig Hospital, Englewood, USA; 9 Spinal Cord Injury and Disability Research, TIRR Memorial Hermann, Houston, USA; 10 Department of Ophthalmology, Feinberg School of Medicine, Northwestern University, Chicago, USA; 11 Department of Medical Social Sciences, Feinberg School of Medicine, Northwestern University, Chicago, USA; 12 Center for Health Services and Outcomes Research, Feinberg School of Medicine, Northwestern University, Chicago, USA; 13 World Health Organization Collaborating Center for the Epidemiology of Musculoskeletal Health and Aging, University of Liege, Liege, Belgium; 14 Present Address: Physical Medicine and Rehabilitation, Feinberg School of Medicine, Northwestern University, Chicago, USA; 15 Present Address: Department of Physical Medicine and Rehabilitation, University of Texas Health Science Center at Houston, Houston, USA; 16 Division of Physical Therapy, Emory University, Atlanta, USA; 17 Health Services Research and Development Service, US Department of Veterans Affairs, Chicago, USA

**Keywords:** Spinal cord injuries, Exoskeleton device, Physical therapy modalities, Gait, Quality-adjusted life years

## Abstract

**Background:**

Few, if any estimates of cost-effectiveness for locomotor training strategies following spinal cord injury (SCI) are available. The purpose of this study was to estimate the cost-effectiveness of locomotor training strategies following spinal cord injury (overground robotic locomotor training versus conventional locomotor training) by injury status (complete versus incomplete) using a practice-based cohort.

**Methods:**

A probabilistic cost-effectiveness analysis was conducted using a prospective, practice-based cohort from four participating Spinal Cord Injury Model System sites. Conventional locomotor training strategies (conventional training) were compared to overground robotic locomotor training (overground robotic training). Conventional locomotor training included treadmill-based training with body weight support, overground training, and stationary robotic systems. The outcome measures included the calculation of quality adjusted life years (QALYs) using the EQ-5D and therapy costs. We estimate cost-effectiveness using the incremental cost utility ratio and present results on the cost-effectiveness plane and on cost-effectiveness acceptability curves.

**Results:**

Participants in the prospective, practice-based cohort with complete EQ-5D data (n = 99) qualified for the analysis. Both conventional training and overground robotic training experienced an improvement in QALYs. Only people with incomplete SCI improved with conventional locomotor training, 0.045 (SD 0.28), and only people with complete SCI improved with overground robotic training, 0.097 (SD 0.20). Costs were lower for conventional training, $1758 (SD $1697) versus overground robotic training $3952 (SD $3989), and lower for those with incomplete versus complete injury. Conventional overground training was more effective and cost less than robotic therapy for people with incomplete SCI. Overground robotic training was more effective and cost more than conventional training for people with complete SCI. The incremental cost utility ratio for overground robotic training for people with complete spinal cord injury was $12,353/QALY.

**Conclusions:**

The most cost-effective locomotor training strategy for people with SCI differed based on injury completeness. Conventional training was more cost-effective than overground robotic training for people with incomplete SCI. Overground robotic training was more cost-effective than conventional training for people with complete SCI. The effect estimates may be subject to limitations associated with small sample sizes and practice-based evidence methodology. These estimates provide a baseline for future research.

## Background

Approximately 18,000 individuals experienced new spinal cord injuries (SCI) in the United States in 2021, and between 253,000 and 378,000 people live with disabilities due to SCI [1]. SCI commonly results in losses of quality and quantity of life due to impairments of bodily control below the level of the lesion. Multiple body systems are affected following a SCI, resulting in wide ranging consequences [2]. At present, the greatest opportunity for improvement is through the preservation and recovery of motor and sensory function below the lesion through a combination of pharmacological and non-pharmacological approaches [3].

### Locomotor training following spinal cord injury

Individuals with SCI commonly have a goal of walking, as they seek to resume active community participation and employment. Because impairments in standing and walking are associated with significant multiple secondary health complications [4], restoration of walking provides physiological and psychosocial benefits [5, 6]. Therefore, locomotor training is a pillar of rehabilitation [7]. Multiple strategies exist to support locomotor training with varying level of evidence, including overground training, treadmill-based training with human assistance, and treadmill-based training with robotic assistance [7]. Overground strategies that use human assistance range from low technology approaches (assistive devices such as LiteGate) to technologically advanced, overhead track-based systems with harnesses and integrated safety features. Treadmill-based strategies typically employ body weight support harnesses while clinicians manually facilitate stepping motions and support trunk control. Human assistance often requires the use of multiple personnel to ensure patient safety and to optimize locomotion, requiring assistance for trunk stability and lower extremity movement. Robotic assistance for locomotor training was introduced to increase stepping repetitions during training without increasing the already high physical exertion required of clinicians [8, 9]. Robotic assistance began with stationary robotic devices that consist of a powered orthosis mounted on a treadmill, but the development of robotic exoskeletons have enabled overground robotic training offering increased environmental flexibiltiy and portability. The evidence base for robotic exoskeleton-assisted therapy is small, but recent findings show a benefit for people with incomplete and complete SCI in terms of self-reported quality of life (i.e., self-reported improvements across multiple domains of general health) [10], and across multiple secondary health outcomes [11–20].

### Value of medical technologies

Assessing the value of a health intervention or medical technology is commonly assessed using a cost-effectiveness analysis (CEA) in health economics. CEAs are always calculated by comparing a new intervention relative to an alternative, reflecting alternative choices for societal resources. In their simplest form, CEAs assess incremental (net) costs and effects. High value therapies are those that can meet one of the following criteria when compared with their alternatives: (1) improve health outcomes and reduce costs, (2) improve health outcomes at a cost that society finds acceptable (a cost that society is ‘willing to pay’), or (3) provide equivalent health outcomes at a lower cost [21].

Despite the high costs of medical technology, there is potential for both health improvement and cost reduction with overground robotic training [22]. Costs following SCI are very high in the first year following injury (direct costs US$523,000), and remain high in subsequent years (US$80,000). On average, SCI will incur direct costs of $3M over a person’s lifespan [23]. Notwithstanding the inclusion of stationary robotic devices, conventional locomotor rehabilitation remains an intensive process with high human capital costs, therefore there is the potential to offset purchase and maintenance costs of overground robotic devices through labor savings. A budget impact analysis of robotic exoskeleton training for those living with SCI found costs to be slightly lower when robotic exoskeleton training is introduced as a locomotor treatment in a health system, however estimates were highly sensitive to clinical utilization patterns [24]. In budget impact analyses, the question of affordability of a new technology is considered within a given jurisdiction (e.g., hospital or health system); however, health effects are not explicitly considered [25]. The lack of cost-effectiveness data remains an economic barrier to technology adoption as it relates to robotic rehabilitation [26]. CEAs can help inform resource allocation, approval, and coverage decisions at the national level, and prioritization of populations who are most likely to benefit from an intervention [27, 28].

The objective of this study was to estimate the cost-effectiveness of overgound robotic training compared with conventional locomotor training for people living with SCI using a practice-based evidence design. This analysis focused exclusively on cost-effectiveness using quality adjusted life years [QALY]) as a health outcome and locomotor training costs. Functional outcomes (i.e., change in walking ability) following locomotor training were not investigated.

## Methods

### Study design and participants

SCI Model Systems are funded by the National Institute on Disability, Independent Living, and Rehabilitation Research (NIDILRR), Administration for Community Living, U.S. Department of Health and Human Services, to support innovation and research in the delivery, demonstration, and evaluation of medical, rehabilitation, vocational, and other health services that meet the needs of people with SCI. Four SCI Model Systems agreed to collaborate on a prospective, longitudinal implementation study to estimate the cost and consequences of locomotor strategies in persons with SCI: (1) The Shirley Ryan AbilityLab (formerly the Rehabilitation Institute of Chicago), (2) Craig Hospital, (3) Shepherd Center, and (4) TIRR Memorial Hermann. Participating Model System sites served as the primary data source for estimates of health-related quality of life and resource utilization in outpatient and community settings. Participating sites collected data prospectively using a practice-based evidence design with the goal of comparing outcomes for people with SCI participating in overground robotic training versus conventional training [29]. Practice-based evidence, “harnesses the complexity of patient and treatment differences in the actual practice of care.” In doing so, it seeks to answer the questions, “Does the treatment work in the real world of everyday practice?” or “For whom does the intervention work best?” [29]. Common features to practice-based evidence design include a pragmatic approach to study inclusion and intervention selection to maximize generalizability, control of patient differences through statistical rather than experimental means, facility and clinic buy in through transdisciplinary teams, and high levels of transparency for all stakeholders. Eligibility criteria for this study were a diagnosis of traumatic SCI, ability to comprehend English, age 18 years or older, and a goal to improve lower extremity function related to gait, balance or functional mobility. Training was not standardized as is typical in practice-based evidence design. All therapists performing overground robotic training received training from the device manufacturers on safe and effective use of the exoskeletons. Overground robotic exoskeletons are not part of conventional practice therefore there was no overlap in interventions.

Individuals with SCI were recruited from a variety of treatment areas including outpatient therapy, activity-based health and wellness centers, and those already participating in other research studies using overground robotic training. Institutional review boards approved the study for all sites.

### Outcomes

#### Quality of life

Participating centers classified SCI level as cervical (C1–7), thoracic (T1–12), lumbar (L1–5), and sacral injury, and used the American Spinal Injury Association Impairment Scale to characterize motor and sensory impairment [30]. For purposes of analysis, we categorized injuries as motor complete or incomplete. Participants completed research instruments before their third session of therapy and after discharge, including the Euro-QoL 5D-3L (EQ-5D) [31].

The primary effectiveness outcome for the economic evaluation was the change in QALYs experienced over the duration of locomotor training using the EQ-5D. The EQ-5D is a generic health measure that assigns a summary value to health profiles [31, 32]. These values facilitate the calculation of QALYs by applying utility weights that convert each EQ-5D health profile into a value on a scale from 0 to 1 where 1 equates to full health and 0 equates to death. The EQ-5D-3L describes 243 unique health profiles and this study applied utility weights derived from representative samples of the UK general public [33] to estimate QALYs [34].

#### Costs

Rehabilitation costs were estimated using the number of rehabilitation minutes multiplied by cost of locomotor training. Locomotor training costs differed per site based on regional human capital costs, use of personnel, and average distribution of locomotor training strategies. Costs are in 2020 United States dollars (USD) and reflect the healthcare perspective, i.e., the costs considered are those that are relevant to decisions made at the level of the health system. We applied rehabilitation costing strategies employed in a previously published budget impact analysis [24] to patient-level resource use. Rehabilitation cost price weights and their sources are shown in Appendix 1.

#### Cost-effectiveness

Cost-effectiveness was calculated as the incremental difference in costs and effects between overground robotic training and conventional training groups. Incremental costs and effects were also analyzed within subgroups of people with complete and incomplete injury. The net benefit static is a helpful way to calculate cost-effectiveness because it rearranges the comparison of cost and effect from ratio to linear values by incorporating the amount that society is willing to pay for an improvement in health. Net benefit was calculated as (50,000 * ∆QALYs) − ∆costs, where $50,000USD represents a conservative threshold for society’s willingness to pay (WTP) for an additional QALY [35, 36]. A positive value for net benefit means that the new treatment is less than the decision-maker’s threshold WTP for an additional QALY. The incremental cost utility ratio was also reported when a therapy produced a larger net effect at a higher net cost allowing us to report a cost per additional QALY gained.

The CEA outcomes are presented graphically on the cost-effectiveness plane and as cost-effectiveness acceptability curves. The cost-effectivenss plane plots the distribution of incremental costs and effects for alternatives. Incremental costs are plotted on the Y-axis and incremental effects are plotted on the X-axis. Differences in costs and effects can fall into one of four quadrants: the upper right quadrant (i.e., northeast) where a therapy increases costs and effects, the lower right quadrant (i.e., southeast) where a therapy decreases costs and increases effects, the lower left quadrant where a therapy decreases costs and decreases effects, and the upper left quadrant where a therapy increases costs and decreases effects. Cost-effectivenss acceptability curves plot the proportion of cost-effectiveness estimates that fall below a budget constraint and illustrate the probability of cost-effectiveness over a range of WTP values [37]. A range of $50,000 to $150,000 USD was used as WTP values to judge cost-effectiveness [36].

### Statistical analysis

Data analyses were conducted in SAS and Excel. We calculated incremental cost utility ratios (ICURs) using a Microsoft Excel (Microsoft, Redmond, WA)-based simulation. The model draws one thousand simulations of cost and effect estimates for multiple treatments under the assumption of multivariate normality [38]. All analyses used the health system perspective. Recognizing injury completeness as a potential confounder [39], our analyses stratified participants by injury severity (motor complete [complete] versus motor incomplete [incomplete]).

## Results

Ninety-nine participants completed the EQ-5D; Table 1 shows sample characteristics. Participants were on average 39 (SD 16) years old, 4.8 (SD 5.27) years from injury to study enrollment, 20% had a complete SCI, 68% were male, and 42% Black/African American. The overground robotic training group was younger, had a greater percentage of White/Caucasian participants (59% vs. 34%), and a greater percentage with complete injuries (28% versus 16%) than the conventional training group.

**Table 1:** Sample characteristics, quality of life, and costs of training strategies

	Full sample N = 99	Conventional training N = 67	Overground robotic training N = 32
Age in years	39 (16)	42 (16)	33 (13)
Sex Males N (%)	66 (66.67)	46 (68.66)	20 (62.50)
Race N (%)			
White or Caucasian	42 (42.42)	23 (34.33)	19 (59.38)
Black or African American	42 (42.42)	35 (52.24)	7 (21.88)
American Indian/Alaska Native	2 (2.02)	1 (1.49)	1 (3.13)
Asian or Pacific Islander	3 (3.03)	1 (1.49)	2 (6.25)
Other, multiracial	9 (9.09)	6 (8.96)	3 (9.38)
Prefer not to answer	1 (1.01)	1 (1.49)	0 (0)
Time (years) from injury to study	4.8 (5.27)	5.07 (4.87)	4.22 (6.05)
Complete injury N (%)	19 (20.21)	10 (16.13)	9 (28.13)
Injury level N(%)			
C1–C8 (%)	39 (41.05)	24 (38.10)	15 (46.88)
T1–T12 (%)	44 (46.32)	29 (46.03)	15 (46.88)
L1–L5 (%)	12 (12.63)	10 (15.87)	2 (6.25)
Unknown	4	4	0
Paraplegia	53 (56.99)	38 (58.46)	15 (53.57)
Tetraplegia	34 (36.56)	21 (32.31)	13 (46.43)
Unknown	6 (6.45)	6 (9.23)	0 (0)
Missing data	6	2	4
EQ-5D utility [0–1]			
Baseline utility	0.39 (0.34)	0.36 (0.34)	0.47 (0.32)
Discharge utility	0.42 (0.36)	0.40 (0.36)	0.48 (0.37)
Therapy minutes	1843 (2522)	1005 (1081)	3598 (3594)
Average cost (standard deviation)	2466 (2835)	1758 (1697)	3952 (3989)

Mean(SD) unless otherwise noted

### Quality of life and costs

On average, both the conventional training and overground robotic training groups reported QALY improvements as measured by EQ-5D Incremental QALY gains were lower and costs were higher for overground robotic training than for conventional training strategies (Table 1). When results were analyzed by injury severity, a different pattern emerged. Table 2 reports disaggregated costs and effects for locomotor training strategy by injury severity. Conventional training resulted in QALY improvements for people with incomplete SCI but not for those with complete SCI. Conversely, overground robotic training resulted in QALY improvements for people with complete SCI but not for those with incomplete SCI. Costs were higher for people with complete versus incomplete SCI. Overground robotic training for those with complete SCI had the highest rehabilitation costs, but was also associated with the largest QALY gains.

**Table 2:** Diaggregated average costs and effects (standard deviation)

	Conventional training		Overground robotic training	
	Incomplete	Complete	Incomplete	Complete
	N = 57	N = 10	N = 23	N = 9
QALYs	0.045 (0.28)	− 0.044 (0.34)	− 0.032 (0.17)	0.097 (0.20)
Costs	1745 (1741)	2450 (1936)	3867 (4529)	4169) (2276)

### Cost-effectiveness

**Fig. 1:**
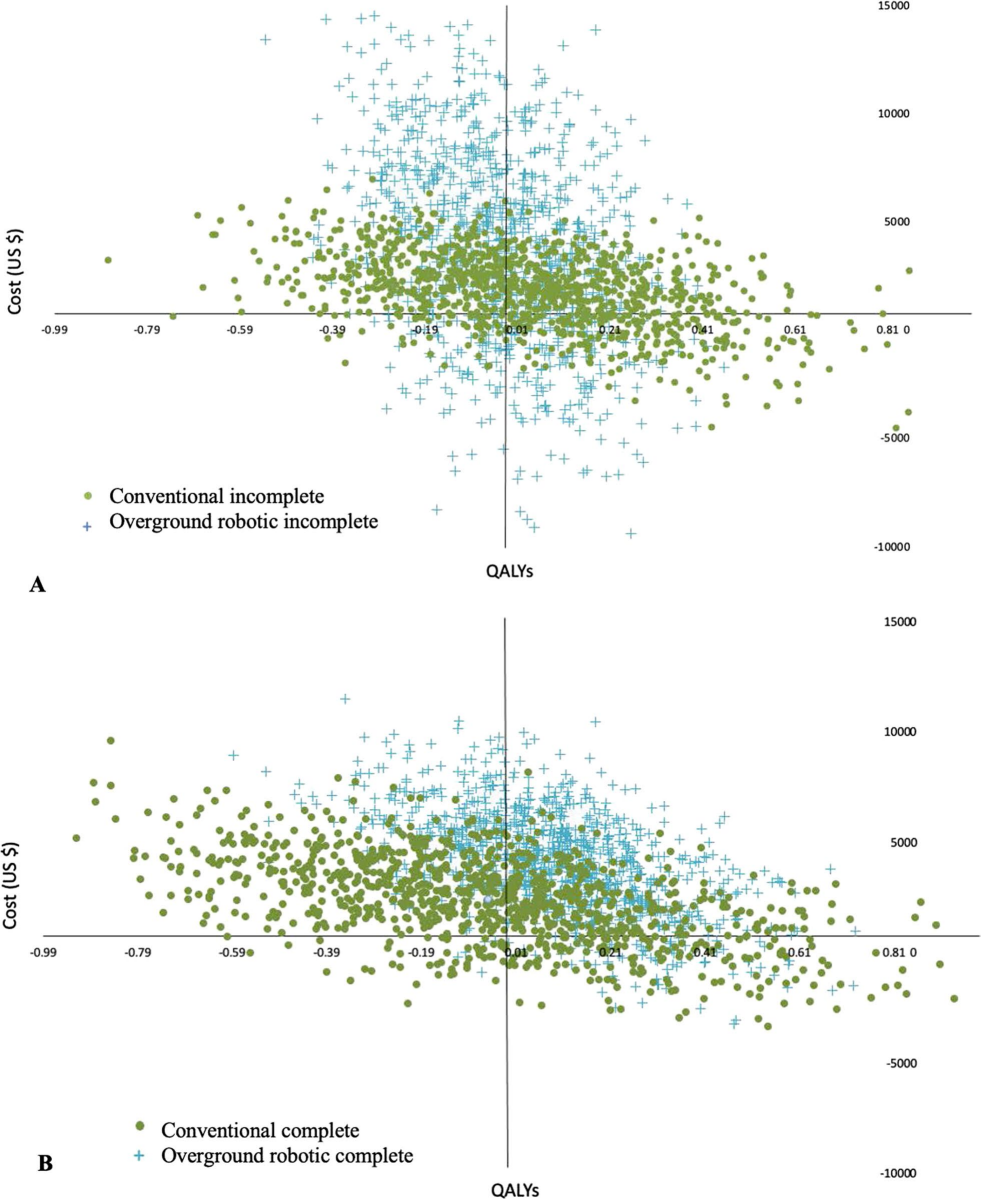
Cost-effectiveness plane of locomotor strategies (conventional and overground robotic) in people with incomplete spinal cord injury (**A**) and complete spinal cord injury (**B**). The cost-effectiveness plane shows four quadrants where an intervention can fall relative baseline. Estimates that fall in the upper right quadrant show strategies that are more effective and cost more, lower right quadrant shows strategies that are more effective and cost less (cost saving), lower left quadrant show strategies that are less effective and costs less, and upper left quadrant show strategies that are less effective and costs more (dominated)

**Fig. 2:**
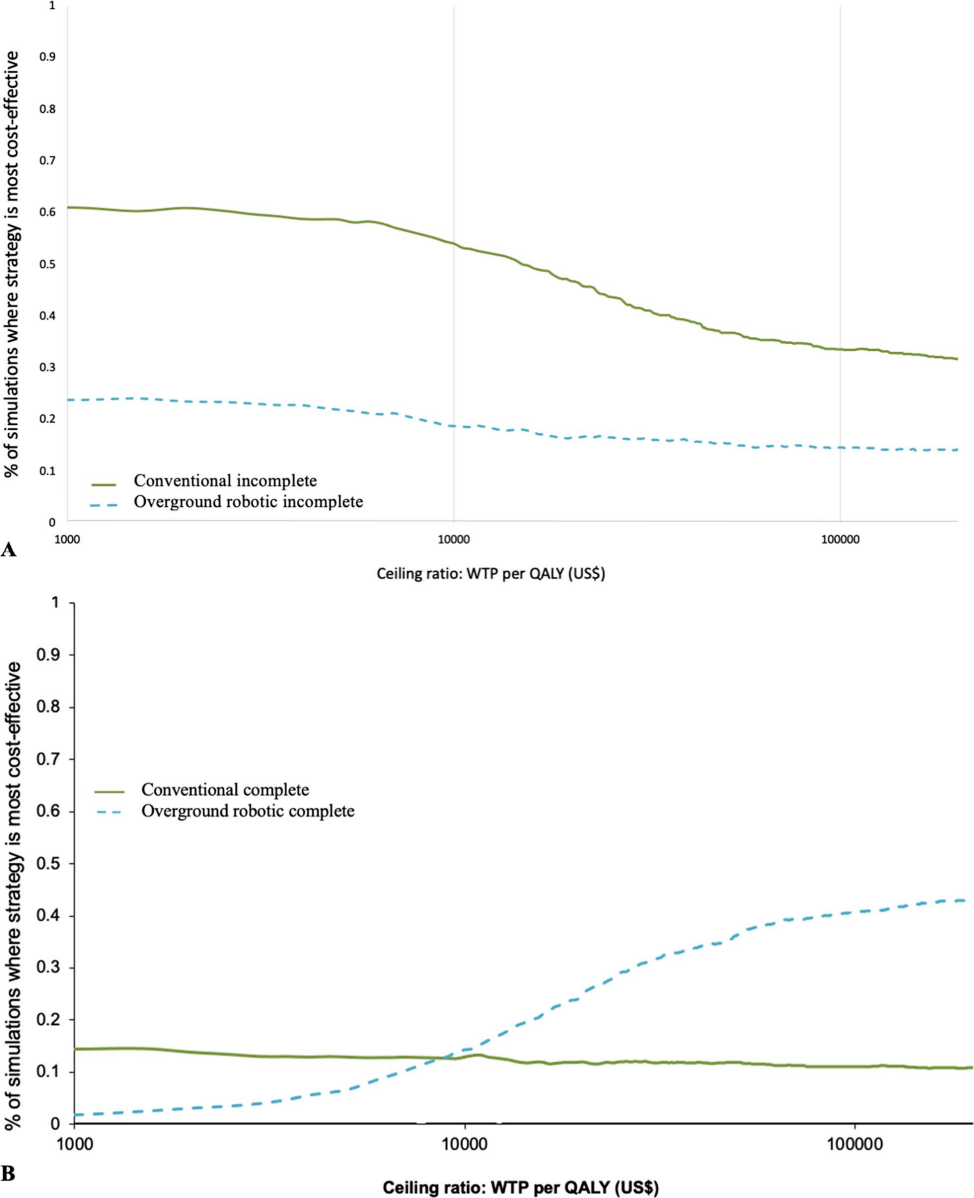
Cost-effectiveness acceptability curve of locomotor strategies (conventional and overground robotic) in people with incomplete spinal cord injury (**A**) and complete spinal cord injury (**B**). The cost-effectiveness acceptability curve presents the percentage of simulations where each strategy is most cost-effective at different levels of willingness to pay (WTP) for an additional quality adjusted life year (QALY)

Our probabilistic CEA produced 1000 simulations to characterize uncertainty of cost and effect parameters in the model [40]. Only conventional training for people with incomplete SCI and overground robotic training for people with complete SCI were estimated to produce a positive net benefit. The cost-effectiveness results are plotted on both the cost-effectiveness plane and presented as cost-effectiveness acceptability curves (Figs. 1 and 2).

#### Incomplete SCI

The majority of cost-effectiveness estimates of conventional strategies for people with incomplete SCI (43%) fall in the right upper quadrant (more effective and more cost), whereas 50% of estimates fall in the left upper quadrant (less effect and more cost) for overground robotic training (Fig. 1A). Conventional training dominated overground robotic training, i.e., it was both more effective and cost less on average. At no level of WTP for an additional QALY was overground robotic training the most cost-effective strategy (Fig. 2A).

#### Complete SCI

Locomotor strategies for people with complete injury show a pattern in the opposite direction (Figs. 1B and 2B). The majority of cost-effectiveness estimates for conventional strategies (52%) fall in the left upper quadrant (less effective and more cost), whereas 65% of estimates for overground robotic training fall in the right upper quadrant (more effective and more cost). With low values of WTP for an additional QALY (<$10,000 USD), conventional locomotor strategies are most cost-effective, however at WTP values greater than $10,000 USD per QALY, overground robotic training showed the greatest probability of cost-effectiveness. When comparing locomotor strategies in people with complete SCI, 75% of simulations showed robotic locomotor training to have the greatest net benefit.

## Discussion

This CEA represents a major contribution to the robotic neurorehabilitation literature and provides key benchmark data for future research. Our analysis used patient-level data from a prospective, practice-based cohort and applied modeling techniques to account for sampling and decision-maker uncertainty. Stratifying our analysis by injury completeness enabled an investigation of the value of locomotor training strategies by clinically meaningful subgroups. We found the most cost-effective locomotor strategy (conventional versus overground robotic) was different in people with incomplete versus complete injury. Conventional locomotor strategies dominated (higher effect and lower cost) overground robotic strategies in the subsample of people with incomplete SCI whereas the most cost-effective strategy for people with complete SCI depends on the the decision-maker’s WTP for an additional year of quality of life. When considered against a conservative WTP value for an additional QALY ($50,000), overground robotic training is cost-effective for people with complete SCI.

Evidence of cost-effectiveness for robotic locomotor training following neurological injury appears to be limited to a single meta-analysis that produced economic estimates for two robotic locomotor training strategies versus conventional locomotor training post stroke [41]. Unfortunately, this CEA has numerous limitations when reviewed against the Consolidated Health Economic Evaluation Reporting Standards checklist [42]. Most crucially, the analysis limited the assessment of uncertainty to a scenario analysis representing different hospital work shifts (5 days versus 6 days with 7.12 and 12 h shifts respectively). It did not address parameter uncertainty (variation in the cost or effect estimates derived from the meta-analysis and costing strategy) or decision maker uncertainty. The decision to apply a single average price weight for both robotic technologies is a limitation because the authors refer to prices for the respective technologies that differ by a factor of 10. Our CEA appears to be the first to assess two competing locomotor strategies for people following SCI using primary data and addressing parameter and decision-maker uncertainty.

QALYs adjust an additional year of life based on the quality of that additional year. Therefore, a treatment that extends life but also produces negative health consequences is given a lower value than a treatment that extends a year of life in perfect health. Historically, the therapy judged to be most cost-effective is often the one that maximizes QALYs within a budgetary constraint. The National Council on Disability recommends against use of the QALY as an outcome for people living with disability because all future years of life are downward-adjusted due to the person’s disability (i.e., they can never attain ‘perfect health’ according to the QALY), making interventions that meet their needs less attractive for funding when compared to interventions among able-bodied populations [43]. An International Society for Pharmacoeconomics and Outcomes Research (ISPOR) Special Task Force defined elements of value that can be added to CEA calculations [21]. The QALY is one of twelve elements of value identified by the ISPOR task group, and was never intended to be used as a single decision-making threshold [44]. We agree that maximizing QALYs while ignoring the populations in which they are produced ignores multiple elements of value important to society. Our analysis provides an important starting point in its use of core value elements identified by the ISPOR task force, QALYs and net costs. Researchers may be interested in using our estimates for benchmark comparison of treatments for people living with disability versus treatments for those who are able-bodied. Future analyses would advance the field by adjusting QALY-estimates to reflect disease severity and equity.

There are concerns that prematurely studying the economic effects of incremental technologies may stall future funding and development [45]. However, these same incremental technologies are being purchased due to hospital market competition that accelerates healthcare equipment expansion. Economic studies often are criticized as coming too early until they are too late [46]. Recognizing this tension between the provision of evidence-based healthcare and optimal timing of technology adoption, our intitial cost analysis of overground robotic technology began with a hypothetical economic model assessing the affordability of introducing these technologies as a locomotor training tool from the perspective of the hospital system [24]. Further research is needed to assess the broad health effects that can be improved via robotic locomotor training and savings that may be produced. Value elements that factor disease severity, the value of adherence-improving factors, and scientific spillovers could increase the understanding of the value added by of overground robotic technology for people with SCI [21]. Should QALYs be more highly weighted in those with more severe SCI? Should incremental technologies that have promise of future innovation have more weight? Does engaging in a novel technology such as overground robotic training increase adherence to locomotor training? Each consideration can theoretically be added to the value framework in conventional CEA, but few examples exist in the literature.﻿

This study adds preliminary evidence of cost-effectiveness using the QALY as an outcome, but generates new questions regarding the incremental value of locomotor strategies in distinct clinical subgroups, i.e., complete versus incomplete SCI. Our results show substantial differences in the QALY estimates for incomplete and complete SCI depending on the locomotor training strategy. In our sample, people with incomplete SCI showed a QALY improvement using conventional training and a QALY loss using overground robotic training; the opposite was true for people with complete SCI. Lower QALYs in the incomplete SCI group using overgound robotic training may be related to differences in physical effort between groups [47], robotic guidance strategies that may not optimally enhance motor learning [48], or a difference between initial expectations and lived experience following training [18]. The greater QALY gain in people with complete SCI using overground robotic training may be related to psychological improvements and the wellness model of health [18, 49]. Additionally, a range of benefits that affect quality of life have been attributed to engaging in robotic locomotor training [50]; however, little evidence exists to suggest a differential effect for complete versus incomplete SCI. A recent randomized trial assessing the effect of overground robotic training on bowel function in people with SCI found overground robotic training may produce a greater improvement in bowel function in people with complete SCI [51]. Because there is no evidence that locomotor training improves walking ability in persons with complete SCI, this subgroup of people with SCI generally do not have access to conventional locomotor training through traditional reimbursement models. Our study suggests that there may be justification to use overground robotic locomotor training with a focus on improving quality of life.

## Strengths and limitations

This study contributes to the literature by working with multiple SCI Model System sites allowing for larger and more geographically diverse study recruitment. We employed simulation using a validated model to address sampling uncertainty and decision-maker uncertainty. We reported on the net benefit of competing interventions and plotted cost-effectiveness findings across multiple levels of WTP per QALY. Our sample was too small to control for level of injury, age, sex, American Spinal Cord Injury Association classification, and other factors that influence rehabilitation outcomes. We attempted to address uncertainty through simulation, but a confounding factor could explain differences that we are reporting.

Locomotor training is intended to build locomotor capacity, but we do not report on measures of walking ability. Clinical practice guidelines to improve locomotor function after incomplete SCI have recently recommended against performing walking interventions using treadmill training with body weight support or exoskeletal robotics on a treadmill or elliptical device to improve walking speed and distance in people greater than 6 months following injury [7]. These strategies were included within the treatment mix that makes up conventional locomotor training [24]. It is possible that those in the conventional locomotor training group would have experienced different outcomes had a protocol-driven treatment for locomotor training been used, however practice-based evidence research designs reflect what actually occurs in clinical practice, not an idealized version of clinical practice. The clinical practice guidelines did not report on overground robotic training in SCI, however a review of wearable exoskeleton robotics for gait training reported improvements with ambulation assessments [52]. We focused exclusively on the EQ-5D as an outcome in this study because of its ability to capture multiple domains of general health and the opportunity to use it as an economic outcome with a generally agreed upon understanding of its value to decision-makers.

We limited the cost inputs to health system rehabilitation costs and the time horizon to one year. Limiting our analysis to health system rehabilitation costs may be acceptable because rehabilitation is the largest cost post-SCI [53], but focusing on health system costs misses potential out of pocket costs borne by individuals and family members. Limiting the analysis to one-year costs may be acceptable because there is no evidence suggesting that incremental differences exist between locomotor training strategies over the long term, though savings have been estimated for locomotor training strategies versus no training [54].

Finally, our study compared two distinct practice models in conventional locomotor training and overground robotic training. Conventional locomotor training was part of an insurance reimbursed rehabilitation model whereas overground robotic training used a combination of self-pay wellness visits and research project participation. The wellness model sessions typically were longer (60 min versus 45 min) and donning and doffing of the robotic exoskeleton added non-therapeutic time (potentially 40 min) [52]. Despite this difference, the overground robotic training group had greater training times. Whereas we account for the time difference in the calculation of costs, we are unable to identify whether the QALY gain for people with complete SCI is attributed to the additional time in overground robotic training or time spent in the wellness model of care itself. This work is preliminary and requires further validation, ideally using one of the following methods—a practice-based evidence design that increases cohort size to allow for greater statistical control, a decision analytic model, or a fully powered RCT with embedded economic analysis.

## Conclusions

The most cost-effective locomotor training strategy for people with SCI differed based on injury completeness. Conventional locomotor training was more cost-effective than overground robotic training for people with incomplete SCI. Overground robotic training was more cost-effective than conventional training for people with complete SCI. The effect estimates may be subject to limitations associated with small sample sizes and practice-based evidence methodology.

## Data Availability

The dataset used and analyzed during the current study are available from the corresponding author upon request.

## Appendix 1

Units costs and associated sources

**Table:**

Component	Cost/unit	Assumptions/sources
*Locomotor devices*^a^		
Overground exoskeleton device	$18.36/session	Capital cost of robot—purchase price ($150,000), annual maintenance contract ($10,000) * 5-year lifespan
Litegait—overground training system	$0.47/session	Rehab hospital purchasing department * 10-year lifespan
body weight supported treadmill and harness system	$6.86/session	Rehabilitation-hospital quality treadmill cost $35,000 + annual maintenance contract ($8,500) * 5-year lifespan
Track-based overground training and harness system	$7.52/session	$225,000 + annual maintenance contract ($7,500) * 20-year life span
Stationary robotic system	$38.95/session	$350,000 + annual maintenance contract ($15,000) * 5-year lifespan
*Personnel*		
PT	$51.58–$58.08	US Bureau of Labor Statistics (BLS) website mean hourly rate * 30% benefits adjusted for regional location of Spinal Cord Injury Model System (SCIMS) center
PT Assistant	$33.66–$43.68	BLS website mean hourly rate * 30% benefits adjusted for regional location of SCIMS center
Exercise specialist	$28.90–$34.46	BLS website mean hourly rate * 30% benefits adjusted for regional location of SCIMS center
PT Aide/tech	$16.76–$20.15	BLS website mean hourly rate * 30% benefits adjusted for regional location of SCIMS center

^a^Device utilization assumptions for calculating per session cost: 7 sessions/day Monday-Friday, 4 on Saturday, 4 on Sunday (with 2 weeks off)—2150 sessions per annum

## Abbreviations

CEA: Cost-effectiveness analysis
ICUR: Incremental cost-utility ratio
ISPOR: International Society for Pharmacoeconomics and Outcomes Research
QALY: Quality-adjusted life years
SCI: Spinal cord injury
USD: United States dollars
WTP: Willingness to pay

## References

[1] National Spinal Cord Injury Statistical Center. Traumatic spinal cord injury facts and figures at a glance birmingham, AL: University of Alabama at Birmingham; 2022.

[2] Krause JS, Saunders LL (2011). Health, secondary conditions, and life expectancy after spinal cord injury. Arch Phys Med Rehabil.

[3] Musselman KE, Shah M, Zariffa J (2018). Rehabilitation technologies and interventions for individuals with spinal cord injury: translational potential of current trends. J Neuroeng Rehabil.

[4] Sezer N, Akkus S, Ugurlu FG (2015). Chronic complications of spinal cord injury. World J Orthop.

[5] Dittuno PL, Ditunno JF (2001). Walking index for spinal cord injury (WISCI II): scale revision. Spinal Cord.

[6] Calhoun CL, Schottler J, Vogel LC (2013). Recommendations for mobility in children with spinal cord injury. Top Spinal Cord Inj Rehabil.

[7] Hornby TG, Reisman DS, Ward IG, Scheets PL, Miller A, Haddad D (2020). Clinical practice guideline to improve locomotor function following chronic stroke, incomplete spinal cord injury, and brain injury. J Neurol Phys Ther.

[8] Krebs HI, Hogan N, Aisen ML, Volpe BT (1998). Robot-aided neurorehabilitation. IEEE Trans Rehabil Eng.

[9] Krebs HI, Volpe BT, Ferraro M, Fasoli S, Palazzolo J, Rohrer B (2002). Robot-aided neurorehabilitation: from evidence-based to science-based rehabilitation. Top Stroke Rehabil.

[10] van Nes IJW, van Dijsseldonk RB, van Herpen FHM, Rijken H, Geurts ACH, Keijsers NLW (2022). Improvement of quality of life after 2-month exoskeleton training in patients with chronic spinal cord injury. J Spinal Cord Med..

[11] Tamburella F, Lorusso M, Tramontano M, Fadlun S, Masciullo M, Scivoletto G (2022). Overground robotic training effects on walking and secondary health conditions in individuals with spinal cord injury: systematic review. J Neuroeng Rehabil.

[12] Chun A, Asselin PK, Knezevic S, Kornfeld S, Bauman WA, Korsten MA (2020). Changes in bowel function following exoskeletal-assisted walking in persons with spinal cord injury: an observational pilot study. Spinal Cord.

[13] Asselin P, Cirnigliaro CM, Kornfeld S, Knezevic S, Lackow R, Elliott M (2021). Effect of exoskeletal-assisted walking on soft tissue body composition in persons with spinal cord injury. Arch Phys Med Rehabil.

[14] Baunsgaard CB, Nissen UV, Brust AK, Frotzler A, Ribeill C, Kalke YB (2018). Exoskeleton gait training after spinal cord injury: an exploratory study on secondary health conditions. J Rehabil Med.

[15] Karelis AD, Carvalho LP, Castillo MJ, Gagnon DH, Aubertin-Leheudre M (2017). Effect on body composition and bone mineral density of walking with a robotic exoskeleton in adults with chronic spinal cord injury. J Rehabil Med.

[16] Faulkner J, Martinelli L, Cook K, Stoner L, Ryan-Stewart H, Paine E (2021). Effects of robotic-assisted gait training on the central vascular health of individuals with spinal cord injury: a pilot study. J Spinal Cord Med.

[17] Ehrlich-Jones L, Crown DS, Kinnett-Hopkins D, Field-Fote E, Furbish C, Mummidisetty CK (2021). Clinician perceptions of robotic exoskeletons for locomotor training after spinal cord injury: a qualitative approach. Arch Phys Med Rehabil.

[18] Kinnett-Hopkins D, Mummidisetty CK, Ehrlich-Jones L, Crown D, Bond RA, Applebaum MH (2020). Users with spinal cord injury experience of robotic Locomotor exoskeletons: a qualitative study of the benefits, limitations, and recommendations. J Neuroeng Rehabil.

[19] Heinemann AW, Kinnett-Hopkins D, Mummidisetty CK, Bond RA, Ehrlich-Jones L, Furbish C (2020). Appraisals of robotic locomotor exoskeletons for gait: focus group insights from potential users with spinal cord injuries. Disabil Rehabil Assist Technol.

[20] Heinemann AW, Jayaraman A, Mummidisetty CK, Spraggins J, Pinto D, Charlifue S (2018). Experience of robotic exoskeleton use at four spinal cord injury model systems centers. J Neurol Phys Ther.

[21] Lakdawalla DN, Doshi JA, Garrison LP, Phelps CE, Basu A, Danzon PM (2018). Defining elements of value in health care-a health economics approach: an ISPOR special task force report [3]. Value Health.

[22] Sorenson C, Drummond M, Bhuiyan KB (2013). Medical technology as a key driver of rising health expenditure: disentangling the relationship. Clinicoecon Outcomes Res.

[23] Miller LE, Herbert WG (2016). Health and economic benefits of physical activity for patients with spinal cord injury. Clinicoecon Outcomes Res.

[24] Pinto D, Garnier M, Barbas J, Chang SH, Charlifue S, Field-Fote E (2020). Budget impact analysis of robotic exoskeleton use for locomotor training following spinal cord injury in four SCI model systems. J Neuroeng Rehabil.

[25] Sullivan SD, Mauskopf JA, Augustovski F, Jaime Caro J, Lee KM, Minchin M (2014). Budget impact analysis-principles of good practice: report of the ISPOR 2012 budget impact analysis good practice II task force. Value Health.

[26] Turchetti G, Vitiello N, Trieste L, Romiti S, Geisler E, Micera S (2014). Why effectiveness of robot-mediated neurorehabilitation does not necessarily influence its adoption. IEEE Rev Biomed Eng.

[27] Drummond MF, Sculpher MJ, Torrance GW, O'Brien BJ, Stoddart GL (2005). Methods for the economics evaluation of health care programmes.

[28] Avancena ALV, Prosser LA (2022). Innovations in cost-effectiveness analysis that advance equity can expand its use in health policy. BMJ Glob Health..

[29] Horn SD, Gassaway J (2007). Practice-based evidence study design for comparative effectiveness research. Med Care..

[30] Roberts TT, Leonard GR, Cepela DJ (2017). Classifications in brief: American Spinal Injury Association (ASIA) impairment scale. Clin Orthop Relat Res.

[31] Devlin N, Parkin D, Janssen B (2020). Methods for analysing and reporting EQ-5D data.

[32] Devlin NJ, Shah KK, Feng Y, Mulhern B, van Hout B (2018). Valuing health-related quality of life: an EQ-5D-5L value set for England. Health Econ.

[33] Rabin R, de Charro F (2001). EQ-5D: a measure of health status from the EuroQol Group. Ann Med.

[34] Mulhern B, Feng Y, Shah K, Janssen MF, Herdman M, van Hout B (2018). Comparing the UK EQ-5D-3L and English EQ-5D-5L value sets. Pharmacoeconomics.

[35] Willan AR (2004). Incremental net benefit in the analysis of economic data from clinical trials, with application to the CADET-Hp trial. Eur J Gastroenterol Hepatol.

[36] Neumann PJ, Cohen JT, Weinstein MC (2014). Updating cost-effectiveness—the curious resilience of the $50,000-per-QALY threshold. N Engl J Med.

[37] Glick HA, Briggs AH, Polsky D (2001). Quantifying stochastic uncertainty and presenting results of cost-effectiveness analyses. Expert Rev Pharmacoecon Outcomes Res.

[38] Barton GR, Briggs AH, Fenwick EA (2008). Optimal cost-effectiveness decisions: the role of the cost-effectiveness acceptability curve (CEAC), the cost-effectiveness acceptability frontier (CEAF), and the expected value of perfection information (EVPI). Value Health.

[39] Garnier-Villarreal M, Pinto D, Mummidisetty CK, Jayaraman A, Tefertiller C, Charlifue S (2022). Predicting duration of outpatient physical therapy episodes for individuals with spinal cord injury based on locomotor training strategy. Arch Phys Med Rehabil.

[40] Briggs AH, Sculpher MJ, Claxton K (2006). Decision modeling for health economic evaluation.

[41] Carpino G, Pezzola A, Urbano M, Guglielmelli E (2018). Assessing effectiveness and costs in robot-mediated lower limbs rehabilitation: a meta-analysis and state of the art. J Healthc Eng.

[42] Husereau D, Drummond M, Augustovski F, de Bekker-Grob E, Briggs AH, Carswell C (2022). Consolidated Health economic evaluation reporting standards 2022 (CHEERS 2022) statement: updated reporting guidance for health economic evaluations. Value Health.

[43] National Council on Disability (2019). Quality-adjusted life years and the devaluation of life with disability: part of the bioethics and disability series.

[44] Kim DD, Basu A (2021). How Does Cost-Effectiveness Analysis Inform Health Care Decisions?. AMA J Ethics.

[45] Boninger M, French J, Abbas J, Nagy L, Ferguson-Pell M, Taylor SJ (2012). Technology for mobility in SCI 10 years from now. Spinal Cord.

[46] Drummond M, Banta D (2009). Health technology assessment in the United Kingdom. Int J Technol Assess Health Care.

[47] Lotze M, Braun C, Birbaumer N, Anders S, Cohen LG (2003). Motor learning elicited by voluntary drive. Brain.

[48] Schmidt RA, Bjork RA (1992). New conceptualizations of practice: common principles in three paradigms suggest new concepts for training. Psychol Sci.

[49] Cahill A, Ginley OM, Bertrand C, Lennon O (2018). Gym-based exoskeleton walking: a preliminary exploration of non-ambulatory end-user perspectives. Disabil Health J.

[50] Shackleton C, Evans R, Shamley D, West S, Albertus Y (2019). Effectiveness of over-ground robotic locomotor training in improving walking performance, cardiovascular demands, secondary complications and user-satisfaction in individuals with spinal cord injuries: a systematic review. J Rehabil Med.

[51] Hubli M, Dietz V, Bolliger M (2012). Spinal reflex activity: a marker for neuronal functionality after spinal cord injury. Neurorehabil Neural Repair.

[52] Rodriguez-Fernandez A, Lobo-Prat J, Font-Llagunes JM (2021). Systematic review on wearable lower-limb exoskeletons for gait training in neuromuscular impairments. J Neuroeng Rehabil.

[53] Munce SE, Wodchis WP, Guilcher SJ, Couris CM, Verrier M, Fung K (2013). Direct costs of adult traumatic spinal cord injury in Ontario. Spinal Cord.

[54] Morrison SA, Pomeranz JL, Yu N, Read MS, Sisto SA, Behrman AL (2012). Life care planning projections for individuals with motor incomplete spinal cord injury before and after locomotor training intervention: a case series. J Neurol Phys Ther.

